# Combined Metabolome and Transcriptome Analysis of Creamy Yellow and Purple Colored *Panax notoginseng* Roots

**DOI:** 10.3390/life13102100

**Published:** 2023-10-23

**Authors:** Muhan He, Guanghui Zhang, Dongfang Huo, Shengchao Yang

**Affiliations:** 1 Office of Academic Affairs, Yunnan Forestry Technological College, Kunming 650224, China; 2013020321@ynftc.edu.cn (M.H.); 2019025961@ynftc.edu.cn (D.H.); 2 State Key Laboratory of Conservation and Utilization of Bio-Resources in Yunnan, The Key Laboratory of Medicinal Plant Biology of Yunnan Province, National & Local Joint Engineering Research Center on Germplasm Innovation & Utilization of Chinese Medicinal Materials in Southwest China, Yunnan Agricultural University, Kunming 650201, China; zgh73107310@163.com

**Keywords:** anthocyanin biosynthesis, ginsenoside, Sanqi, saponins, terpenoid biosynthesis

## Abstract

*Panax notoginseng* (Burk.) F.H. Chen is a species of the Araliaceae family that inhabits southwestern China, Burma, and Nepal. It is cultivated on a commercial scale in Yunnan province, China, owing to its significance in traditional Chinese medicine. *Panax notoginseng* roots are usually yellow-white (HS); however, purple roots (ZS) have also been reported. The majority of *P. notoginseng* research has concentrated on the identification and production of natural chemicals in HS; however, there is little to no information about the composition of ZS. Using UPLC-MS/MS, we investigated the global metabolome profile of both ZS- and HS-type roots and discovered 834 metabolites from 11 chemical groups. There were 123 differentially accumulated metabolites (DAM) in the HS and ZS roots, which were classified as lipids and lipid-like molecules, polyketides, organoheterocyclic chemicals, and organooxygen compounds. We investigated the associated compounds in the DAMs because of the importance of anthocyanins in color and saponins and ginsenosides in health benefits. In general, we discovered that pigment compounds such as petunidin 3-glucoside, delphinidin 3-glucoside, and peonidin-3-O-beta-galactoside were more abundant in ZS. The saponin (eight compounds) and ginsenoside (26 compounds) content of the two varieties of roots differed as well. Transcriptome sequencing revealed that flavonoid and anthocyanin production genes were more abundant in ZS than in HS. Similarly, we found differences in gene expression in genes involved in terpenoid production and related pathways. Overall, these findings suggest that the purple roots of *P. notoginseng* contain varying amounts of ginsenosides and anthocyanins compared to roots with a creamy yellow color.

## 1. Introduction

*Panax notoginseng* (Burk.) F.H. Chen is a species of Panax in the Araliaceae family. It is found in southern China, Burma, and Nepal. The majority of commercially available *P. notoginseng* comes from the Chinese province of Yunnan, specifically from the city of Wenshan [1,2]. *P. notoginseng* root, often known as Sanqi or Tianqi in East Asian countries, is an important herb in Traditional Chinese Medicine (TCM). It has been widely used as a tonic and hemostatic medication for over 400 years, and it still continues to play an important role in TCM [3]. With the increased study of TCM, considerable efforts have been made to investigate the phytochemistry and pharmacological effects of *P. notoginseng*, with over 200 chemicals identified. These components include saponins, amino acids and their derivatives, phytosterols, flavonoids, and polysaccharides. Among these, the most active components are saponins [3,4]. A variety of pharmacological effects, such as cardioprotective, neuroprotective, antitumor, anti-aging, and anti-inflammatory effects, as well as protection against cerebrovascular injury, hemostasis and anticoagulation, and diabetes mellitus, of these compounds have been discovered. The promising progress on *P. notoginseng*’s health benefits necessitates a timely and comprehensive evaluation of our current understanding of the composition of different variants within this species.

Anthocyanins are glycosylated polyphenolic chemicals with a range of colors in flowers, seeds, fruits and vegetative tissues. Because anthocyanins are water-soluble pigments found predominantly in cell vacuoles, the intravacuolar environment influences their hue or color quality. The amount of anthocyanin in the tissue is determined by the balance between biosynthesis and degradation [5]. The anthocyanin biosynthesis pathway is a branch of the flavonoid pathway. It involves several genes that are part of the flavonoid biosynthetic pathway. These genes are divided into early and late biosynthesis genes (EBGs and LBGs, respectively). The EBGs are chalcone synthase (CHS), chalcone isomerase (CHI), and flavanone 3-hydroxylase (F3H). These are common flavonoid biosynthetic genes and are involved in the biosynthesis of downstream flavonoids. Their expression is not consistently related to anthocyanin biosynthesis [6]. The LBGs include flavonoid 3′-hydroxylase (F3′H) or flavonoid 3′,5′-hydroxylase (F3′5′H), which produce dihydroflavonols. Meanwhile, dihydroflavonol 4-reductase (DFR) converts the three dihydroflavonols to colorless leucoanthocyanidins, which are then transformed into colorful anthocyanidins by anthocyanidin synthase (ANS). Finally, several members of the glycosyltransferase enzyme family, such as flavonoid 3-O-glucosyltransferase (UFGT), bind sugar molecules to anthocyanidins and can be acylated with aromatic acyl groups by acyltransferases. Of these, F3′H and F3′5′H are the major enzymes responsible for anthocyanin color diversity by influencing their B-ring hydroxylation pattern, whereas DFR substrate specificity affects anthocyanin composition and pigmentation [5,6]. Few studies have reported EBGs, LBGs, and transcription factors in *P. notoginseng* in purplish-green aerial stems [7,8]. However, the mechanism of the differential coloration of *P. notoginseng* roots with normal creamy and purplish color has not been investigated.

The biosynthesis of ginsenoside begins with the creation of the skeleton, for which (3S)-2,3-oxidosqualene is the primary precursor. Both the mevalonate and methyl-D-erythritol phosphate routes are engaged in ginsenoside biosynthesis, with the former playing a larger role. The creation of squalene biosynthesis, which includes squalene epoxidases, oxidosqualene cyclases, and cytochrome p450s (CYPs), follows. Ginsenoside biosynthesis also includes UDP-sugar biosynthesis, in which UGTs play essential roles [9]. Most ginsenoside triterpene scaffolds are glycosylated by UGTs, which involves UDP-sugars as sugar donors to generate various ginsenosides. Ginsenoside sapogenins are additionally ornamented at the C-3 or/and C-20 hydroxyl groups of PPD-type saponins, C-6 or/and C-20 hydroxyl groups of PPT-type saponins, and C-3 hydroxyl or/and C-28 carboxyl groups of OA-type saponins. This third stage determines ginsenoside structural diversity [9]. The genome sequencing of both ginseng [10] and notoginseng revealed a genome-scale metabolic network and a comprehensive perspective of ginsenoside production [11,12]. The major steps involved in ginsenoside and saponin biosynthesis in these two species are well elaborated, which can help us understand the differential regulation of the natural variants.

Organ colors might differ between cultivars of the same species. According to one study, the *P. ginseng* varieties registered in Korea and China differ in terms of their features. Notably, some cultivars have contrasting root and stem colors, such as Gumpoong, which has a creamy root hue and a purple stem color [13]. A natural variation of the genotype “Sanqi” was discovered during our ongoing research activities, whose stem base was purple as opposed to green/dark green. The roots were purple on the inside, unlike the regular plants of this kind, which have creamy yellow roots. However, the possible mechanisms for the different coloration are not known. Advances in omics have greatly improved our understanding of biological systems in plants [9]. Therefore, an integrated strategy combining a non-targeted metabolomics approach and transcriptome sequencing was utilized in this study to find chemical variations and associated transcriptome signatures across root samples of the same genotype with two different colors.

## 2. Material and Methods

### 2.1. Reagents

The reagents used for LC-MS/MS analysis were purchased from Sigma Aldrich (Sigma-Aldrich Shanghai Trading Co., Ltd., Shanghai, China) (ammonium acetate, ammonium hydroxide) and Merck (acetonitrile).

### 2.2. Plant Material and Sample Preparation

**Figure 1:**
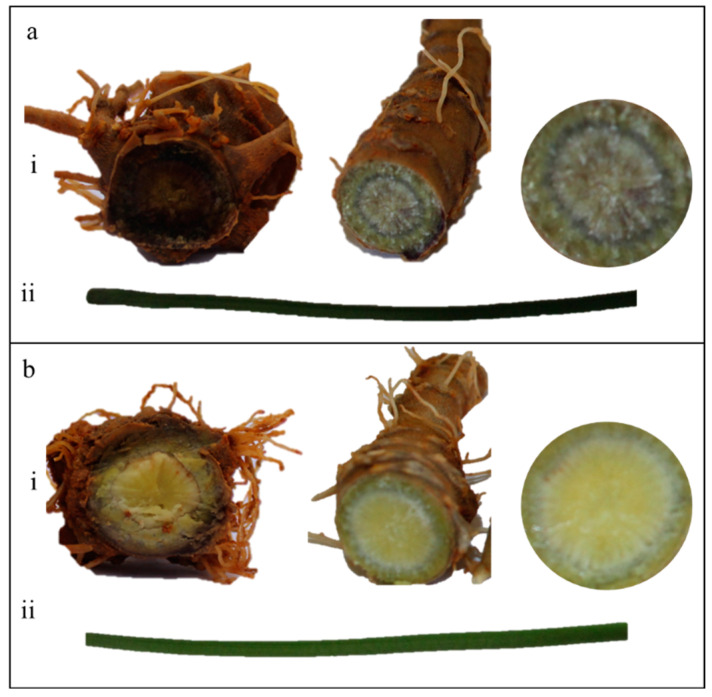
(**a**) Natural variant and (**b**) standard variety “Sanqi” (i) root and (ii) stem cuttings showing differences in color.

Tap roots (yellow-white—HS and purple—ZS) of three-year-old plants of the *P. notoginseng* variety “Sanqi” growing in Yanshan County, Wenshan Prefecture, China, were sampled in September 2022 (Figure 1). The tap roots were rinsed with distilled water, immediately frozen in liquid nitrogen, and stored at −80 °C. The samples were collected in triplicate. The plant tissues (80 mg) were ground into a fine powder using a mortar and pestle. To the homogenized solution, 1000 mL of methanol/acetonitrile/H2O (2:2:1, *v*/*v*/*v*) was added for metabolite extraction, followed by centrifugation for 15 min at 14,000 *g* at a low temperature of 4 °C. The supernatant was a vacuum centrifuge. The samples were redissolved in 100 L of acetonitrile/water (1:1, *v*/*v*) solvent for LC-MS analysis.

### 2.3. Global Metabolome Analysis

Analyses were carried out in Shanghai Applied Protein Technology Co., Ltd. (Shanghai, China) utilizing an ultra-performance liquid chromatography (UHPLC, 1290 Infinity LC, Agilent Technologies, Santa Clara, CA, USA) linked to a quadrupole time-of-flight (AB Sciex TripleTOF 6600). The samples were separated using an Agilent 1290 Infinite UHPLC on a C-18 column at 40 °C. The injection volume was 2 mL, and the flow rate was set to 0.4 mL/min. The mobile phase A contained 25 mM ammonium acetate and 0.5% formic acid in water, while mobile phase B contained methanol. The following was the gradient elution procedure: 0–0.5 min, 5% B; thereafter, B increased linearly from 0.5 to 10 min; 10–12.0 min, B remained at 100%; B changed linearly from 100% to 5% from 12.0 to 12.1 min; from 12.1 to 16 min, B remained at 5%. Throughout the analysis, the material was kept at 4 °C in an automated sampler. A random sequence was employed for sample analysis in order to avoid the influence produced by instrument fluctuation. QC samples were placed in the sample queue to monitor and assess the stability and dependability of the data.

The ESI source conditions were as follows: Ion Source Gas1 was set to 60, Ion Source Gas2 was set to 60, curtain gas was set to 30, source temperature was set to 600 °C, and IonSpray Voltage Floating was set to 5500 V. The instrument was set to acquire over the *m*/*z* range 60–1000 Da in mass spectrometry (MS) alone, and the accumulation time for the TOF MS scan was set at 0.20 s/spectra. The instrument was set to acquire over the *m*/*z* range of 25–1000 Da in auto MS/MS acquisition, and the accumulation time for the product ion scan was set at 0.05 s/spectra. The information-dependent acquisition mode with high sensitivity was used to acquire the product ion scan. The following parameters were set: the collision energy was set to 35 V with 15 eV; the declustering potential was set to 60 V (+) and 60 V (−); isotopes inside 4 Da were excluded; and the number of candidate ions to monitor per cycle was set to 10.

ProteoWizard MSConvert was used to convert the raw MS data (wiff.scan files) to MzXML files before importing them into the freely available XCMS program. The following parameters were utilized for peak selection: the centWave *m*/*z* value was 10 ppm, the peak width was c (10,60), and the prefilter was c (10,100). Peak grouping parameters were bw = 5, mzwid = 0.025, and minfrac = 0.5. Collection of Algorithms of MEtabolite pRofile Annotation was used for isotope and adduct annotation. Only variables with more than 50% of nonzero measurement values in at least one group were retained in the retrieved ion features. Metabolite identification was accomplished by comparing the accuracy *m*/*z* value (10 ppm) and MS/MS spectra to an in-house database developed using accessible, authentic standards.

### 2.4. Statistical Analysis of Metabolome Data

The processed data were subjected to multivariate data analysis, including Pareto-scaled principal component analysis (PCA) and orthogonal partial least-squares discriminant analysis (OPLS-DA), after being adjusted to total peak intensity. The model’s robustness was assessed using 7-fold cross-validation and response permutation testing. Each variable’s VIP (variable significance in the projection) value in the OPLS-DA model was calculated to indicate its contribution to categorization. Metabolites with VIP values greater than one were subjected to a univariate Student’s *t*-test to determine the significance of each metabolite; *p* values less than 0.05 were considered statistically significant.

### 2.5. Transcriptome Sequencing

Total RNAs were isolated from 50 mg of individual root samples using RNeasy Plant Mini Kit (Qiagen, Seoul, Korea) following the manufacturer’s instructions. The purity of the extracted RNAs was determined using 1% agarose gels and a NanoPhotometer spectrophotometer (IMPLEN, Los Angeles, CA, USA). We used a Qubit RNA Assay Kit in a Qubit 2.0 Fluorometer (Life Technologies, Carlsbad, CA, USA) to quantify RNA. The RNA Nano 6000 Assay Kit of the Agilent Bioanalyzer 2100 system (Agilent Technologies, Santa Clara, CA, USA) was also used to test RNA integrity. Sequencing libraries were prepared using the NEB Next Ultra RNA Library Prep Kit according to the manufacturer’s instructions [14] and sequenced on an Illumina HiSeq 2000 platform.

Clean reads were obtained by removing low-quality reads. We then used Trinity for de novo transcriptome assembly [15]. The unigenes were then annotated in different databases, including NR [16], Swiss-Prot [17], KEGG [18], and KOG, using Blastx (e-value < 0.00001). The unigene expression level was computed as reads per kilobase per million mapped reads (RPKM) [19]. Differentially expressed genes (DEGs) were screened if the log2 fold change was >1 or <−1 with an FDR value ≤ 0.05 between the different types of roots. Next, the DEGs were enriched into the KEGG pathways [20]. Heatmaps of key DEGs were produced with values of log2 fold change values in TBtools [21].

## 3. Results

### 3.1. Global Metabolome Profile

**Figure 2:**
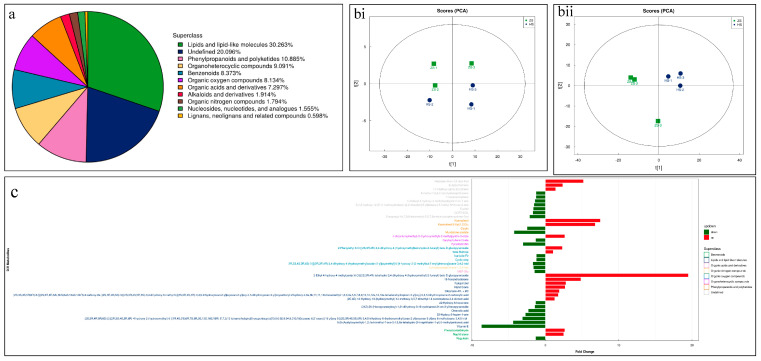
(**a**) Classification and % content of the detected metabolites in HS and ZS roots. (**b**) Principal component analyses of metabolites detected in HS and ZS roots in (i) negative and (ii) positive ion modes. (**c**) Highly up- and down-accumulated metabolites in HS compared to ZS.

Global metabolome analysis using UPLC-MS/MS resulted in the identification of 834 metabolites classified into 11 superclasses (Figure 2a). Lipids and lipid-like compounds were the highest percent of the detected metabolites, followed by undefined compounds, phenylpropanoids and polyketides, organoheterocyclic compounds, and benzenoids. The least detected compounds were classified as lignans, neolignans, and related compounds (Figure 2a). The PCA plots showed that the replicates for each root type tended to group together (Figure 2b).

We considered the compounds differentially accumulated metabolites (DAMs) if the log2 FC was higher than +1 and lower than −1. This resulted in the screening of 59 up- and 64 down-accumulated metabolites in HS compared to ZS (Table S1). The up-accumulated DAMs in HS were mostly classified as flavonoids and isoflavonoids, prenol lipids, organonitrogen compounds, steroids and derivatives, benzene and substituted derivatives, pyridines and derivatives, and others (Table S1). Contrarily, the DAMs that were up-accumulated in ZS belonged to lipids and lipid-like molecules (prenol lipids and fatty acids), phenylpropanoids and polyketides (flavonoids, isoflavonoids, coumarins and derivatives, cinnamic acids, and diarylheptanoids), organoheterocyclic compounds (benzopyrans, indoles and derivatives, and tetrapyrroles and derivatives), and organooxygen compounds (carbohydrates and conjugates) (Table S1). These results highlight that the two types of notoginseng roots differ in their metabolomic profiles.

The highest log2 FC (ZS vs. HS) was noted for 4-hydroxybenzoylcholine, followed by 3-ethyl-4-hydroxy-4-methylpentyl 6-O-[(2S,3R,4R)-tetrahydro-3,4-dihydroxy-4-(hydroxymethyl)-2-furanyl]-beta-D-glucopyranoside, 1-(tert-butylamino)-3-[[4-(morpholin-4-yl)-1,2,5-thiadiazol-3-yl]oxy]propan-2-ol, cyanidin 3-O-glucoside, kaempferol, kaempferol 5-Xyl(1,2)Glc, and luteoline. Notably, we observed higher contents of kaempferol 7-O-glucoside, orientanol E, fisetin, 19-norandrosterone, quinolactacin A, and many others. Conversely, the compounds that had the lowest log2 FC were isorhamnetin, petunidin 3-glucoside, 2-[5-[2-[2-[5-(2-oxopropyl)oxolan-2-yl]propanoyloxy]butyl]oxolan-2-yl]propanoic acid, vitamin E, 6-(beta-D-glucopyranosyloxy)-4,5,6,7,8,8a-hexahydro-5-hydroxy-5-(1-hydroxy-1-methylethyl)-3,8-dimethyl-2(1H)-azulenone, 5,7,3′-trihydroxy-6,4′,5′-trimethoxyflavone, and 1,7-bis(4-hydroxyphenyl)-3-heptanyl 6-O-[(2S,3R,4R)-3,4-dihydroxy-4-(hydroxymethyl)tetrahydro-2-furanyl]-beta-D-glucopyranoside. Other notable compounds that showed lower accumulation in HS compared to ZS include bussein, coumarin, quercetin, tributylamine, loliolide, gardneramine, pyraclostrobin, naadp, ligustilide, and alantrypinone (Figure 2c).

### 3.2. Differential Flavonoid Contents in HS and ZS

In total, there were fifteen DAMs classified as flavonoids (phenylpropanoids and polyketides), nine of which were present in higher quantities in HS compared to ZS, whereas six others showed the opposite accumulation trends (Table 1). Nine of these were flavonoid glycosides, while the rest were flavones. The highest accumulated content was noted for kaempferol 5-xyl (1,2)Glc, followed by kaempferol and kuwanone H in HS. HS had higher quantities of pigment compounds such as astragalin, kuwanone H, cyanin, fisetin, kaempferol 7-O-glucoside, luteoline, kaempferol 5-Xyl (1,2)Glc, and cyanidin 3-O-glucoside. On the contrary, ZS had higher quantities of isorhamnetin, petunidin 3-glucoside, quercetin, delphinidin 3-glucoside, peonidin-3-O-beta-galatoside, and cirsimarin, indicating that both natural mutants vary in their flavonoid glycoside and flavones content. The presence of higher quantities of petunidin 3-glucoside, delphinidin 3-glucoside, and peonidin-3-O-beta-galactoside could be a possible reason for the purple colour in ZS.

### 3.3. Differential Prenol Lipid and Saponin Contents in HS and ZS

Seven and fourteen prenol lipids showed higher and lower accumulation in HS and ZS, respectively (Table 1). The prenol lipids that were present in higher quantities in HS included diterpenoids, terpene lactones, sesquiterpenoids, and triterpenoids. The ZS roots, in addition to these compound classes, also include quinone and hydroquinone lipids and hopanoids.

Eight compounds annotated as saponins were detected in the roots of the two natural variants. Soyasaponin Ba, saikosaponin C, and saikosaponin A were present at higher levels in HS. The others, including saponarin, chikusetsusaponin Iva, chikusetsusaponin IV, licoricesaponin G2, and licoricesaponin H2, showed higher contents in ZS. The sum of intensities of all the saponins was higher in ZS than in HS (Table 1).

There were 26 compounds annotated as ginsenosides (Table 2). Although these compounds did not accumulate differentially (except ginsenoside Rk1), owing to their importance, we explored differences in their accumulation patterns in the two root types. Eighteen of these ginsenosides were present in higher quantities in ZS than HS, whereas eight were present in higher quantities in HS. The highest content in ZS was detected for ginsenoside Rk1, followed by Rb2, XLIX, Rg2, and notoginsenoside R1. On the other hand, ginsenoside Rg3, Rg6, Rg5, F2, and Rh1 were accumulated in higher quantities in HS compared to ZS. The sum of intensities of all metabolites showed that ZS had higher ginsenoside content compared to HS.

### 3.4. Differential Contents of Alkaloids and Derivatives, Carbohydrates, and Organoheterocylcic Compounds in HS and ZS

There were only three DAMs classified as alkaloids and derivatives. These three alkaloids, i.e., alkergot (1305-fold), vinca (1.64-fold), and reserpine (5.5-fold), were present in higher quantities in HS compared to ZS. Three and five carbohydrates and conjugates were accumulated in higher quantities in HS and ZS, respectively (Table S1). Particularly, we observed that HS had higher quantities of fructose compared to ZS.

We observed that eleven and ten organoheterocyclic compounds were present in relatively higher quantities in HS and ZS, respectively. The compounds detected in higher quantities in HS included quinolactacin A, bupirimate, amethopterin, and furaquinocin C. Compounds that accumulated in higher quantities in ZS included thionine cation, biliverdin, deoxykhivorin, alantrypinone, ligustlide, and pyraclostrobin (Table S1).

### 3.5. Differential Transcriptome Profile of HS and ZS Roots

Transcriptome analysis revealed the expression of 55,773 unigenes, of which 12,153 were DEGs. In total, 458 and 1641 DEGs were exclusively expressed in ZS and HS, respectively. Of all the DEGs, 2736 and 9417 showed higher and lower expressions in ZS and HS, respectively, compared to each other. This is consistent with the metabolome profile-based observation that a relatively higher number of metabolites showed lower accumulation patterns in ZS compared to HS (Table S2). We specifically focused on the differential expression of DEGs enriched in flavonoid, anthocyanin, and terpenoid biosynthesis pathways, considering their important role in anthocyanin biosynthesis. Additionally, we also looked for the differential gene expression of the genes enriched in starch and sucrose biosynthesis pathways.

#### 3.5.1. Differential Expressions of Genes Enriched in Flavonoid and Anthocyanin Biosynthesis Pathways

**Figure 3:**
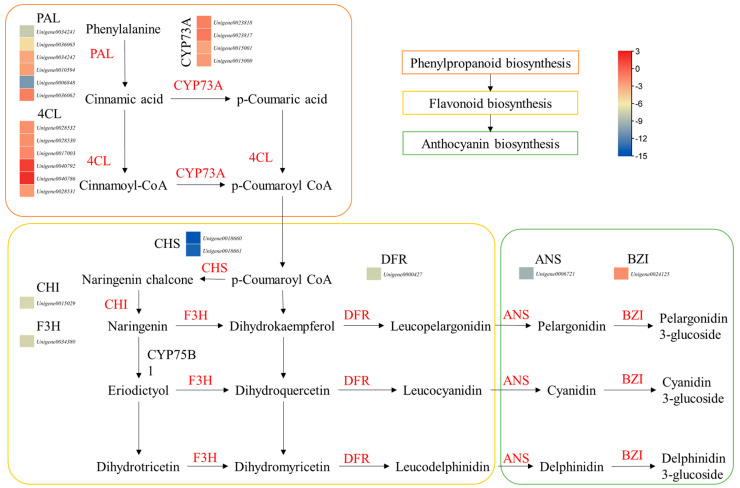
Differential expression of genes enriched in phenylpropanoid, flavonoid, and anthocyanin biosynthesis pathways. The heatmaps show log2 FC values of the DEGs (HS vs. ZS). The genes given in red are differentially expressed. PAL, phenylalanine ammonia lyase; 4CL, 4-coumarate:CoA ligase; CYP73A, cytochrome P450 CYP73A100; CHS, chalcone synthase; CHI, chalcone isomerase; F3H, flavonoid 3-hydroxylase; DFR, dihydroflavonol reductase; ANS, leucoanthocyanidin dioxygenase 1; BZI, anthocyanin 3-O-galatosyltransferase.

There were 17, 1, and 3 DEGs enriched in the flavonoid biosynthesis, anthocyanin biosynthesis, and flavone and flavonol biosynthesis pathways, respectively. Interestingly, all the DEGs enriched in flavonoid biosynthesis pathways had higher expressions in ZS compared to HS. The DEGs included CHI, CHS, C4H, cytochrome p450s, DFR, flavanone 3-dioxygenase isoform 1, flavonoid-3′-hydroxylase, flavonol synthase, and others (Figure 3; Table S2). These upregulations were also consistent with the higher expressions of genes enriched in the phenylalanine biosynthesis pathway. Finally, we observed that the genes enriched in the anthocyanin biosynthesis pathway were also expressed higher in ZS compared to HS. These results clearly indicate that the coloration of the ZS root is due to higher anthocyanin biosynthesis, which is possibly due to the relatively higher expressions of the associated genes.

#### 3.5.2. Differential Expression of Genes Enriched in Terpenoid Biosynthesis-Related Pathways

**Figure 4:**
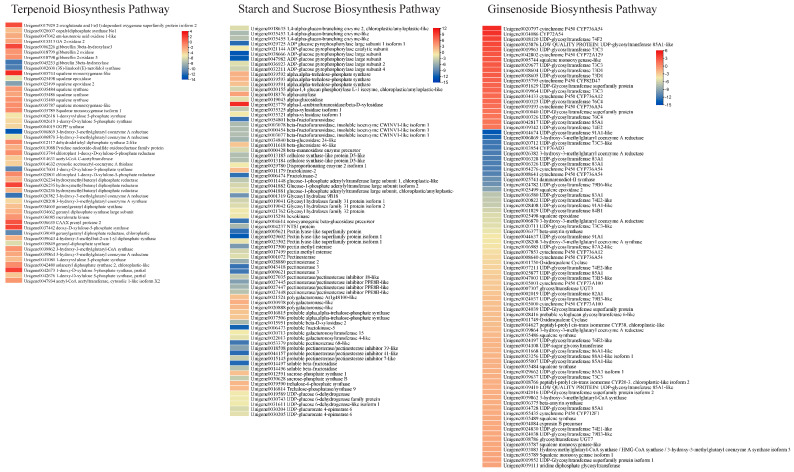
Heatmaps of differentially expressed genes enriched in terpenoid biosynthesis pathways (mono-, di-, tri-, sesquiterpenoids, and terpenoid backbone), starch and sucrose biosynthesis pathways, and ginsenoside biosynthesis pathway. The heatmaps express log2 fold change values. The unigene names are followed by KEGG annotation of the genes.

Considering the metabolome profile results that mono-, di-, tri-, and sesquiterpenoids (including ginsenosides and saponins) were differentially accumulated between the two types of roots, we explored the expression changes in related genes. There were 1, 8, 32, and 8 DEGs enriched in monoterpenoid, diterpenoid, terpenoid, and sesquiterpenoid biosynthesis pathways, respectively. The gene enriched in monoterpenoid biosynthesis, i.e., (3S)-linalool/(E)-nerolidol synthase, had higher expression in HS than in HS. Four of the eight diterpenoid biosynthesis enriched genes were gibberellin related, i.e., two gibberellin 3-beta-hydroxylases and two gibberellin 2 oxidases. Of these, one gibberellin 3-beta-hydroxylase had higher expression in HS than ZS. Conversely, the others had higher expressions in ZS compared to HS. These genes were enriched in the gibberellin-related part of the diterpenoid biosynthesis pathway. The gene copalyldiphosphate synthase No1 (*Unigene0020607*) had higher expression in ZS, suggesting that the genes present in the upstream pathway, i.e., terpenoid backbone biosynthesis, could be responsible for the changes in expression of these genes. We also found that 32 DEGs were enriched in the terpenoid backbone biosynthesis pathway. Most of the DEGs enriched in this pathway showed similar expression trends as those enriched in mono- and di-terpenoid biosynthesis pathways, i.e., they had higher expressions in ZS compared to HS (Figure 4). Thus, it is possible that the higher expressions of the DEGs enriched in terpenoid biosynthesis and related pathways are responsible for the higher detected content of terpenoids as well as saponins. Moreover, we also studied the expression changes in the ginsenoside biosynthesis-related genes [22] and observed that 3-hydroxy-3-methylglutaryl coenzyme A reductase, 3-hydroxy-3-methylglutaryl coenzyme A synthase, beta-amyrin synthase, and several other squalene biosynthesis-related transcripts had lower expressions in ZS compared to HS. Conversely, several transcripts of CYP736A54s, CYP72A129, CYP82D47, CYP36A12, UDP-glycosyltransferase 74F2, 85A1-like, 73C3, 73D1, 76C4, and 74E2 showed higher expressions in ZS compared to HS (Figure 4).

#### 3.5.3. Differential Expression of Genes Enriched in Starch and Sucrose Biosynthesis Pathways

A relatively larger number of DEGs (77) were enriched in starch and sucrose biosynthesis pathways, 15 of which had higher expression in HS, while the rest had higher expressions in ZS. The genes that had lower expression in HS were mostly related to trehalose biosynthesis (alpha-trehalose-phosphate synthase), D-galacturonate biosynthesis (polygalacturonase-like), and dextrin biosynthesis (alpha-amylase). Conversely, those having higher expression in ZS were mostly enriched in key steps involved in D-xylose, D-galaxturonate, starch/glycogen, sucrose, and α-D-glucose biosynthesis or associated interconversions (Figure 4). The observation that both roots differed in the accumulation of metabolites classified as carbohydrates is consistent with the expression changes in a large number of DEGs. This clearly indicates that the two roots have different carbohydrate contents.

## 4. Discussion

*Panax notoginseng* is an important member of the Araliaceae family, which has long been used in TCM. It contains a number of bioactive compounds, including triterpenes (saponins), amino acids, polyacetylenes, phytosterols, flavonoids, and polysaccharides. These pharmacologically active compounds are useful in the treatment of multiple diseases [3,23]. We had found a natural variant of *P. notoginseng* var. Sanqi, which has a purple stem base as well as roots (Figure 1). Considering the earlier work on different colored ginseng (*P. ginseng*) [24], we hypothesized that the newly found variant could also have a different metabolomic profile. The global metabolome profile of the roots of both normal and purple root color variants indicated that both have different metabolome compositions. Firstly, the presence of 11 compound classes in both types is consistent with the earlier work on the metabolome profiles of *P. notoginseng* in that the two are rich in health-beneficial compounds [2,24,25]. The differential accumulation of the 123 metabolites between the two types of roots with different colors indicates that both can offer variable contents of a range of compounds. Earlier works have shown that *Panax* species differ in their metabolomic profiles [25,26]. Within one species, the different tissues of the same plant can also offer variations in the contents of a range of compounds [27]. However, the presence of differential content of compounds in the same variety and tissue can be attributed to natural variations [28].

Differences in plant tissues have been reported in various plants, such as duckweed, where mutants showed significant differences in anthocyanin and proanthocyanin content compared to the wild type [29]. The authors found that these differences were mainly attributed to higher expressions of genes enriched in flavonoid and anthocyanin biosynthesis pathways. Similarly, our observations that the ZS roots had a purplish color and had higher contents of pigment compounds associated with these colors, i.e., delphinidin-3-O-glucoside (which gives blue shades in plants [30]), peonidin-3-O-beta-galactoside (cherry red hue [31]), and petunidin-3-glucoside (purplish colors [32]). These colors could be the most likely reason for the purplish color of the ZS roots. The relatively higher content of these compounds, together with the higher expressions of the important anthocyanin biosynthesis EBG as well as LBGs, are possible causes for the different root color in HS and ZS (Figure 3; Tables S1 and S2). Differences in EBG and LBG expression in different plants have been associated with differences in anthocyanin biosynthesis and resulting color differences. For example, comparative transcriptome analysis of sweet potato [33], potato [34], and Chinese red radish [35] indicated that the quantities of the anthocyanins in roots of different cultivars are due to respective changes in EBG and LBG expressions. Moreover, the higher expressions of genes involved in mono-, di-, tri-, and sesquiterpenoids in ZS and consistent accumulations of terpenoids also propose that these genes are expressed highly, which convert terpenoids into downstream metabolites (Table 1; Figure 4). Similar observations have been made in anthocyanin and terpenoid biosynthesis in *Curcuma alismatifolia* [36]. This is understandable because these pathways are present upstream of the flavonoid and anthocyanin biosynthesis pathways [37,38].

Next, the contrasting content of the multiple saponins and ginsenosides in HS vs. ZS (Table 1 and Table 2) and consistent expression changes in genes enriched in terpenoid (monoterpenoid, diterpenoid, terpenoid, and sesquiterpenoid) biosynthesis pathways (Figure 4) indicate that multiple terpenoid biosynthesis pathways are involved in the differential accumulation of these metabolites [39]. The saponin biosynthesis starts from the mevalonate pathway, and expression changes in the downstream pathway cause significant variation in saponin accumulation in different tissues of plants [40]. Combined metabolome and transcriptome studies in *Artemisia argyi* showed that the differences in saponins were due to expression changes in terpenoid biosynthesis and associated pathways [41]. Other studies on *Bacopa monnieri,* [42], ginseng [43], *Entada phaseoloides,* [44], *Trillium govanianum* [45], and many other plants revealed that the expression changes in these pathways result in differential accumulation/biosynthesis of saponins [46]. The tetracyclic terpenoids are the major types of ginsenosides and have been characterized as the main bioactive compounds of the *Panax* species. Their biosynthesis involves several major enzymes such as 3-hydroxy-3-methylglutaryl coenzyme a reductase, farnesyl pyrophosphate synthase, squalene synthase, squalene epoxidase, dammarenediol-ii synthase and β-amyrin synthase, CYP450S, and UGTs [22]. The results that the transcripts associated with genes showed contrasting gene expressions in the two types of *P. notoginseng* roots explains the variable accumulation of the saponins and ginsenosides in HS and ZS. Finally, the expression changes in large number of starch and sucrose biosynthesis genes and differential accumulation of related compounds in the two types of roots is consistent with the earlier findings that they are co-expressed with the terpenoid biosynthesis genes [2,24,25].

## 5. Conclusions

Global metabolome profiling of *P. notoginseng* roots with different colors i.e., creamy yellow and purple, showed that they differ in their metabolomic profile. The key different metabolite classes are flavonoids, isoflavonoids, coumarins and derivatives, cinnamic acids, diarylheptanoids, carbohydrates and conjugates, terpenoids (saponins and ginsenosides), benzopyrans, indoles and derivatives, and tetrapyrroles and derivatives. Transcriptome sequencing showed that the purple coloration is possibly due to the higher expression of EBGs and LBGs enriched in flavonoid and anthocyanin biosynthesis pathways. In comparison, the differences in the saponin and ginsenoside content could be due to the expression differences in the genes enriched in terpenoid backbone biosynthesis and mono-, di-, tri-, and sesquiterpenoid biosynthesis pathways. Overall, these results open several windows for future research on the composition and biosynthesis of health-beneficial compounds in the studied root variants of *P. notoginseng*.

## Figures and Tables

**Table 1:** Differentially accumulated flavonoids and terpenoids in HS and ZS.

Name	ZS	HS	VIP
Flavonoids			
Astragalin	29,285.41	58,855.67	0.31
Kuwanone H	175,924.72	380,343.06	0.85
Cyanin	30,834.59	114,356.61	0.57
Fisetin	4772.81	23,764.34	0.27
Kaempferol 7-O-glucoside	6665.64	35,655.17	0.34
Luteolin	27,684.04	167,846.96	0.75
Kaemoferol 5-Xyl(1,2)Glc	371,313.56	2,522,548.76	2.94
Kaempferol	60,197.24	451,163.88	1.26
Cyanidin 3-O-glucoside	16,118.21	121,586.82	0.65
Isorhamnetin	122,855.36	10,537.55	0.74
Petunidin 3-glucoside (redness)	100,899.18	8745.49	0.67
Quercetin (yellow)	111,426.07	28,178.06	0.64
Delphinidin 3-glucoside (blue)	23,123.91	8462.79	0.27
Peonidin-3-O-beta-galactoside	23,483.30	9733.83	0.27
Cirsimarin	98,019.10	43,608.94	0.54
Terpenoids			
(1S,7R,8S,13R,16S)-11-Ethyl-7,16-dihydroxy-13-methyl-6-methylidene-11-oxido-11-azoniahexacyclo [7.7.2.15,8.01,10.02,8.013,17]nonadecan-4-one	11,211,216.71	26,520,502.70	7.11
Puberanidine	20,943.91	51,010.89	0.32
Songorine	708,045.81	1,760,762.82	1.88
Costunolide	23,576.27	64,661.15	0.38
2-(Hydroxymethyl)-6-(6-hydroxy-6-methyl-3-propan-2-ylcyclohex-3-en-1-yl)oxyoxane-3,4,5-triol	116,793.89	329,721.54	0.88
1,2,3,7,8,8a-Hexahydro-8,8a-dimethyl-alpha-methylene-7-oxo-, methyl ester, (2R,8R,8aR)-2-naphthaleneacetic acid	59,043.07	180,697.48	0.67
Alisol B	16,855.61	71,937.26	0.46
Vitamin E	2,299,499.25	263,429.96	3.16
6-(beta-D-Glucopyranosyloxy)-4,5,6,7,8,8a-hexahydro-5-hydroxy-5-(1-hydroxy-1-methylethyl)-3,8-dimethyl-2(1H)-azulenone	78,032.41	9429.80	0.57
5-[5-(Acetyloxymethyl)-1,2,4a-trimethyl-7-oxo-3,4,8,8a-tetrahydro-2H-naphthalen-1-yl]-3-methylpentanoic acid	499,205.13	112,607.45	1.39
Jaslanceoside B	6988.78	1927.01	0.16
Methyl 2-[(1R,5R,6R,13S,14S,16S)-14-acetyloxy-6-(furan-3-yl)-1,5,15,15-tetramethyl-8,17-dioxo-7-oxatetracyclo [11.3.1.02,11.05,10]heptadec-10-en-16-yl]acetate	25,525.21	7318.93	0.30
Methyl (4S,5Z,6S)-5-(2-acetyloxyethylidene)-4-[2-[2-(4-hydroxyphenyl)ethoxy]-2-oxoethyl]-6-[(2S,3R,4S,5S,6R)-3,4,5-trihydroxy-6-(hydroxymethyl)oxan-2-yl]oxy-4H-pyran-3-carboxylate	13,986.65	4650.23	0.22
22-Hydoxy-2-hopen-1-one	3,007,955.76	1,101,922.68	3.15
Thalictoside VI	37,078.70	15,368.85	0.30
Oleanolic acid	1,441,725.01	616,186.60	2.09
Fasciculic acid B	242,430.23	104,663.11	0.82
gamma-Tocotrienol	100,671.78	44,365.08	0.55
4-[3-(Beta-D-glucopyranosyloxy)-4-hydroxy-2,6,6-trimethyl-1-cyclohexen-1-yl]-2-butanone	36,243.32	16,285.50	0.32
4-[4-(beta-D-Glucopyranosyloxy)-2-hydroxy-2,6,6-trimethylcyclohexylidene]-3-buten-2-one	175,514.13	81,705.56	0.70
Methyl (2R)-2-[(1S,3S,7R,8R,9R,12S,13R)-13-(furan-3-yl)-6,6,8,12-tetramethyl-17-methylidene-5,15-dioxo-2,14-dioxatetracyclo [7.7.1.0(1),(1)(2).0(3),]heptadecan-7-yl]-2-hydroxyacetate	35,298.09	16,864.56	0.32
Soyasaponin Ba	350,981.37	556,773.96	0.68
Saikosaponin C	14,398.34	17,367.60	0.17
Saikosaponin A	7169.80	7361.35	0.07
Saponarin	7923.95	7336.98	0.08
Chikusetsu saponin IVa	59,988.31	46,791.18	0.33
Chikusetsusaponin IV	1,001,468.09	778,420.57	1.30
Licoricesaponin G2	7037.42	4891.18	0.15
Licoricesaponin H2	7100.14	4900.68	0.14

**Table 2:** List of ginsenosides detected in HS and ZS roots and their relative intensities.

Name	Formula	ZS	HS	VIP
Ginsenoside Rk1	C42H70O12	189,659	78,964	0.767
Ginsenoside Rb2	C53H90O22	223,897	148,380	0.772
Ginsenoside Rb3	C53H90O22	4946	3397	0.114
Gypenoside XLIX	C52H86O21	143,124	108,313	0.498
Ginsenoside Rg2	C42H72O13	43,202,337	33,761,551	8.485
Notoginsenoside R1	C47H80O18	20,990	17,045	0.170
Ginsenoside Re	C48H82O18	253,494	208,204	0.446
Ginsenoside Ro	C48H76O19	122,893	101,173	0.395
Ginsenoside-Rg1	C42H72O14	3,033,671	2,548,503	1.874
Ginsenoside Rb1	C54H92O23	219,216	184,301	0.511
Ginsenoside F3	C41H70O13	13,315	11,361	0.132
20(R)-Ginsenoside Rg2	C42H72O13	6,017,154	5,217,599	2.454
Ginsenoside Rf	C42H72O14	484,261	421,190	0.675
Ginsenoside F1	C36H62O9	121,104	110,746	0.352
Ginsenoside Rd	C48H82O18	197,316	185,131	0.334
2′(R)-Ginsenoside-Rg3	C42H72O13	889,560	850,039	0.583
Ginsenoside RG1	C42H72O14	299,515,744	287,639,124	3.734
Ginsenoside Rh2 (S-FORM)	C36H62O8	774,036	769,964	0.540
Ginsenoside compound K	C36H62O8	49,263	51,108	0.193
Ginsenoside Rh3	C36H60O7	5,414,310	5,803,840	1.205
Ginsenoside-Rb2	C53H90O22	164,788	179,290	0.465
Ginsenoside Rh1	C36H62O9	10,660	11,688	0.085
Ginsenoside F2	C42H72O13	279,103	319,577	0.758
Ginsenoside Rg5	C42H70O12	67,297	77,097	0.420
Ginsenoside Rg6	C42H70O12	2,426,696	2,821,872	2.225
Ginsenoside Rg3 (R-FORM)	C42H72O13	17,677	21,474	0.192

## Data Availability

The raw RNA-seq data have been submitted to NCBI SRA under the project number PRJNA983978.

## Supplementary Materials

The following supporting information can be downloaded at: https://www.mdpi.com/article/10.3390/life13102100/s1, Supplementary Table S1. Differentially accumulated metabolites in HS and ZS roots of *Panax notoginseng*.; Supplementary Table S2. Differentially expressed genes enriched in phenylpropanoid, flavonoids, and anthocyanin biosynthesis pathways.
